# Developmental and Activity-Dependent miRNA Expression Profiling in Primary Hippocampal Neuron Cultures

**DOI:** 10.1371/journal.pone.0074907

**Published:** 2013-10-03

**Authors:** Myrrhe van Spronsen, Eljo Y. van Battum, Marijn Kuijpers, Vamshidhar R. Vangoor, M. Liset Rietman, Joris Pothof, Laura F. Gumy, Wilfred F. J. van IJcken, Anna Akhmanova, R. Jeroen Pasterkamp, Casper C. Hoogenraad

**Affiliations:** 1 Cell Biology, Faculty of Science, Utrecht University, Utrecht, The Netherlands; 2 Department of Neuroscience, Erasmus Medical Center, Rotterdam, The Netherlands; 3 Department of Neuroscience and Pharmacology, Brain Center Rudolf Magnus, University Medical Center Utrecht, Utrecht, The Netherlands; 4 Department of Cell Biology and Genetics, Erasmus Medical Center, The Netherlands; 5 Center for Biomics, Erasmus Medical Center, Rotterdam, The Netherlands; University of Edinburgh, United Kingdom

## Abstract

MicroRNAs (miRNAs) are evolutionarily conserved non-coding RNAs of ∼22 nucleotides that regulate gene expression at the level of translation and play vital roles in hippocampal neuron development, function and plasticity. Here, we performed a systematic and in-depth analysis of miRNA expression profiles in cultured hippocampal neurons during development and after induction of neuronal activity. MiRNA profiling of primary hippocampal cultures was carried out using locked nucleic-acid-based miRNA arrays. The expression of 264 different miRNAs was tested in young neurons, at various developmental stages (stage 2–4) and in mature fully differentiated neurons (stage 5) following the induction of neuronal activity using chemical stimulation protocols. We identified 210 miRNAs in mature hippocampal neurons; the expression of most neuronal miRNAs is low at early stages of development and steadily increases during neuronal differentiation. We found a specific subset of 14 miRNAs with reduced expression at stage 3 and showed that sustained expression of these miRNAs stimulates axonal outgrowth. Expression profiling following induction of neuronal activity demonstrates that 51 miRNAs, including miR-134, miR-146, miR-181, miR-185, miR-191 and miR-200a show altered patterns of expression after NMDA receptor-dependent plasticity, and 31 miRNAs, including miR-107, miR-134, miR-470 and miR-546 were upregulated by homeostatic plasticity protocols. Our results indicate that specific miRNA expression profiles correlate with changes in neuronal development and neuronal activity. Identification and characterization of miRNA targets may further elucidate translational control mechanisms involved in hippocampal development, differentiation and activity-depended processes.

## Introduction

The hippocampus is a limbic system structure in the medial temporal lobe of the brain that plays an essential role in learning and memory in animals and humans. During brain development, hippocampal pyramidal neurons originate from hippocampal neuroepithelial cells and dentate granular progenitors and undergo typical neurodevelopmental stages involving neuronal polarization, axon outgrowth, dendritogenesis, synapse formation, and maturation of synaptic function. In fully differentiated hippocampal neurons, electrophysiological studies have demonstrated the existence of activity-dependent synaptic plasticity such as long term potentiation (LTP) and long-term depression (LTD), which is thought to play a key role in the refinement of neuronal circuitry and considered to be the cellular correlate of learning and memory [1], [2], [3]. Despite the significance of the hippocampus in forming new memories, our understanding of gene regulation mechanisms that underlie neuronal development and synaptic plasticity is quite limited. Post-transcriptional mechanisms, such as alternative mRNA splicing, mRNA trafficking and translational control are believed to play an important role in the regulation of neuronal gene expression [4], [5], [6]. Now it is becoming increasingly clear that the microRNA pathway also has an important impact on neuronal development, survival, function, and plasticity [7], [8], [9].

MicroRNAs (miRNAs) are a class of approximately 22 nucleotides long non-coding RNAs that regulate mRNA expression at the posttranscriptional level through mRNA degradation or translational repression. To date, hundreds of miRNAs have been identified in mammalian genomes and they are predicted to target one-third of all genes in the genome, where each miRNA is expected to target around 100–200 transcripts [10], [11]. The central nervous system is a rich source of miRNA expression [12], [13], [14], with a diversity of miRNA functions that affect many neuronal genes. A large number of miRNAs have been identified in the brain [12], [13], [14], [15], [16], [17], [18] including several miRNAs that are specifically expressed in glia cells and neurons [14], [19], [20]. Recent studies have shown that miRNA function is essential for the development of the zebrafish nervous system [21] and plays a role in neuronal plasticity in the rodent brain [7], [9]. Conditional knockout of the miRNA biosynthetic enzyme Dicer in the developing mouse brain has demonstrated that miRNAs have a critical role in neuronal survival in various brain regions [22], [23], [24], [25], including the hippocampus [26], [27], [28], [29]. Likewise, disruption of Dicer at later time points suggests that alterations in miRNA expression are associated with the degeneration of mature neurons in mice [30], [31]. Others have shown that miRNAs can play fundamentally important roles in more specific neurobiological processes such as proliferation, differentiation, neurite growth and apoptosis [8], [32], [33], [34]. A growing number of reports have revealed that deregulation of miRNA expression contributes to several human neurological, psychiatric and neurodegenerative diseases [27], [35], [36], [37], [38] (. Recent data showed that loss of Dicer and failure of mature miRNA expression may be a feature of hippocampal sclerosis in patients with temporal lobe epilepsy [39].

Like in many other brain regions, miRNAs play vital roles in hippocampal survival, development, function and plasticity [26], [27], [28], [29]. Selective inactivation of Dicer during hippocampal development causes abnormal neuronal morphology and affects the number of hippocampal progenitors [29] and results in an array of phenotypes including reduced dendritic branching, and large increases in dendritic spine length in mature neurons [26]. Using microarray, real-time RT-PCR, in situ hybridization, and deep-sequencing approaches, several studies have identified miRNAs in the hippocampus during development or in adulthood [15], [18], [40], [41], after induction of neuronal activity [42], [43], [44] or injury [45], [46]. While most of these studies where performed in hippocampal brain tissue or slices, in this study we focus on primary rat hippocampal neurons in culture and analyze the miRNA expression profiles during development and after induction of neuronal activity. We make use of the classical and well-studied primary hippocampal neuron culture system as developed by Banker and collaborators [47], [48]. Hippocampal cells are isolated from late stage rat embryos and can be grown for weeks on glass coverslips in serum-free medium. One particular advantage of this culture system is that cells in the embryonic hippocampal tissue become apparently resynchronized upon isolation and subsequently differentiates in culture in a reproducible way along five morphologically well-defined stages [47], [49]. The hippocampal cells become polarized, develop extensive axonal and dendritic arbors and form functional synaptic connections within a week after plating the cells [47]. For detailed analysis of synaptic plasticity mechanisms and dendritic spine morphology the hippocampal neurons are maintained in culture for a longer time (over >2–3 weeks). Many labs have used hippocampal neuronal cultures to study the expression and subcellular distribution of proteins or mRNAs at specific developmental stages [50], [51] and address fundamental questions in neuronal polarity, spine development and synaptic plasticity [52], [53], [54], [55], [56].

To systematically profile miRNA expression in cultured hippocampal neurons during development and after induction of neuronal activity we have used locked nucleic-acid-based miRNA arrays that contain probes for 264 different miRNAs. The purpose of this study was to (1) profile miRNAs expressed in primary cultured rat hippocampal neurons, (2) identify specific miRNAs expression patterns during neuronal development, (3) identify miRNAs induced by NMDA receptor-dependent synaptic plasticity (chemical LTP and LTD protocols) and (4) identify miRNAs induced by prolonged changes in global network activity (homeostatic plasticity protocols). We also performed functional assays with a subset of miRNAs, which showed reduced expressions levels at stage 3 neurons. Sustained expression of 13 of these miRNAs during early neuronal development accelerates axonal outgrowth. These data indicate that decreased expression of miRNAs during neuronal polarization is possibly involved in suppressing axon outgrowth at early stages of development.

## Results

### Timing of hippocampal neuron differentiation in vitro

**Figure 1:**
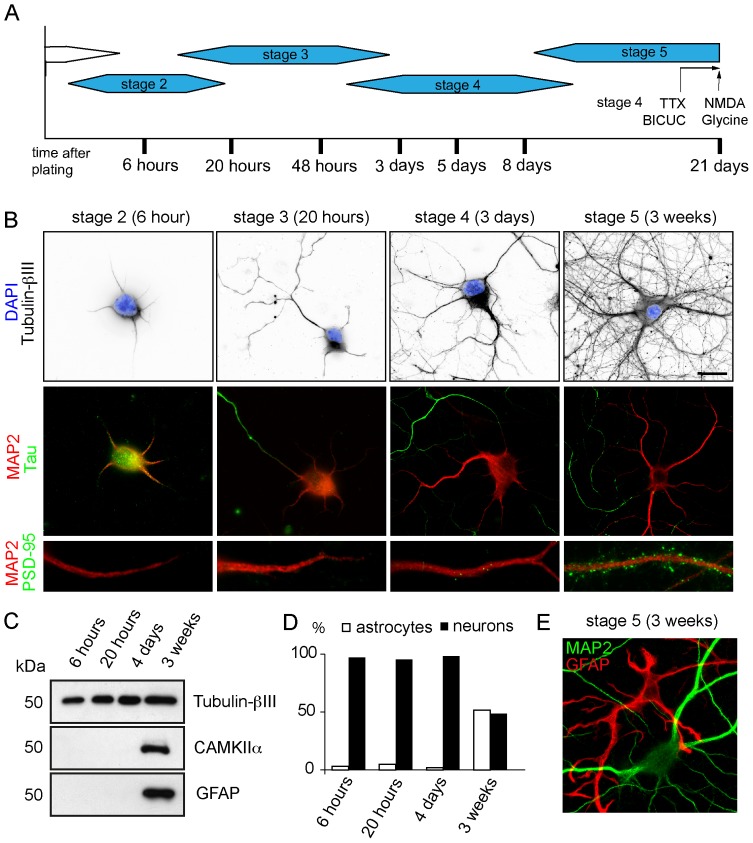
Timing of the stages of neuronal differentiation. A) Schematic overview of different stages of the development of hippocampal neurons in culture. Developing hippocampal neurons were harvested at seven indicated time points at basal state and following the induction of neuronal activity using different chemical stimulation protocols. Synaptic activity was blocked with the voltage-gated sodium channel blocker tetrodotoxin (TTX, 2 μM, 48 h) or enhanced with the GABAA receptor antagonist bicuculline (Bicuc, 40 μM, 48 h). Bath application of 50 μM NMDA for 5 min induces “chemical” long-term depression (LTD) and activation of synaptic NMDARs with 200 μM glycine for 5 min triggering “chemical” long-term potentiation (LTP) in cultured neurons. B) Representative images of hippocampal neurons in culture, fixed at the indicated times and stained for the nucleus (DAPI), neuron specific tubulin (tubulin-ßIII), axon marker Tau, dendrite specific protein MAP2 and postsynaptic protein PSD-95. Scale bar, 20 μm C) Western blot analysis of extract from hippocampal neurons in culture for 6 hours (stage 2), 20 hours (stage 3), 4 days (stage 4) and 3 weeks (stage 5) using indicated antibodies. D) Quantification of the percentage of MAP2 and GFAP positive cells in hippocampal neurons culture for 6 hours (stage 2), 20 hours (stage 3), 4 days (stage 4) and 3 weeks (stage 5). Note that in hippocampal culture of 3 weeks, ∼50% of cells are GFAP positive. E) Representative images of hippocampal neurons in culture, fixed at 3 weeks and stained for dendrite specific protein MAP2 and glial fibrillary acidic protein (GFAP), a marker for astrocytes.

To establish comprehensive miRNA expression profiles during the development of primary rat hippocampal neurons in culture, seven time points were chosen between 6 hours and 21 days in culture. These time points correspond to the peak periods for which major cellular and physiological events occur during neuronal development in culture as defined by Dotti and Banker [47], [48] (Fig. 1A, B). Shortly after plating, cells have a symmetric appearance and extend lamella all around the cell body (stage 1). After 6 hours in culture the cells display initial outgrowth of minor neurites (Fig. 1, stage 2); the growth cones at the tips of the neurites are highly motile and neurites extend and retract over short distances. Then, one neurite grows for an extended period of time without retracting and acquires axonal characteristics. The axon is identified in most neurons by staining for the axon-specific marker tau at 20–48 h in the culture (Fig. 1, stage 3). The axon continues to grow rapidly, whereas the remaining neurites elongate more slowly and become dendrites. At the end of stage 3 many axonal growth cones reached neighbouring neurons and the remaining minor neurites acquire characteristics of dendrites and are positive for the dendrite-specific marker MAP2 (Fig. 1). At 5–8 days (stage 4), the cells have a greater number of outgrowing and branching dendrites and the first synapses are formed. Within the next days, the dendrites of pyramidal neurons form dendritic protrusions/spines and an extensive network of synaptic connections. After 12 days in culture (stage 5), all cells are fully developed, display a mature neuronal morphology and the dendrites show clear punctate PSD-95 staining, representing postsynaptic contacts (Fig. 1). At 21 days synaptic connectivity has been consolidated and the neurons are considered to be fully mature.

The difference with the original Banker protocol [47], [48] is that we use serum-free neurobasal medium supplemented with B27 but do not treat the cultures with AraC on DIV3, so that dividing non-neuronal cells, typically astrocytes will expand in long-term cultures [57]. The major advantage of this method is that at later stages of development, the glial cell help to promote neuronal survival and enhance synapse formation and synaptic transmission [58], [59]. To determine the potential contribution of astrocytes miRNAs on the profiling experiments, we quantify the relative abundance of astrocytes and neurons in the cultures at different time points during development using Western blot analysis (Figure 1C) and immunohistochemistry (Figure 1D, E). These experiments demonstrate that neurons are the predominant population after 6 hours, 20 hours and 4 days in culture (stage 2–4) but that astrocytes are abundantly present after 3 weeks (stage 5). At 3 weeks, an equal number of neurons and astrocytes is present in the cultures (Figure 1D, E). The mixture of miRNAs from the different cell types at this stage should be taken into account during the interpretation of the miRNA expression profiles.

### miRNAs expression profiling of mature hippocampal neurons in culture

**Figure 2:**
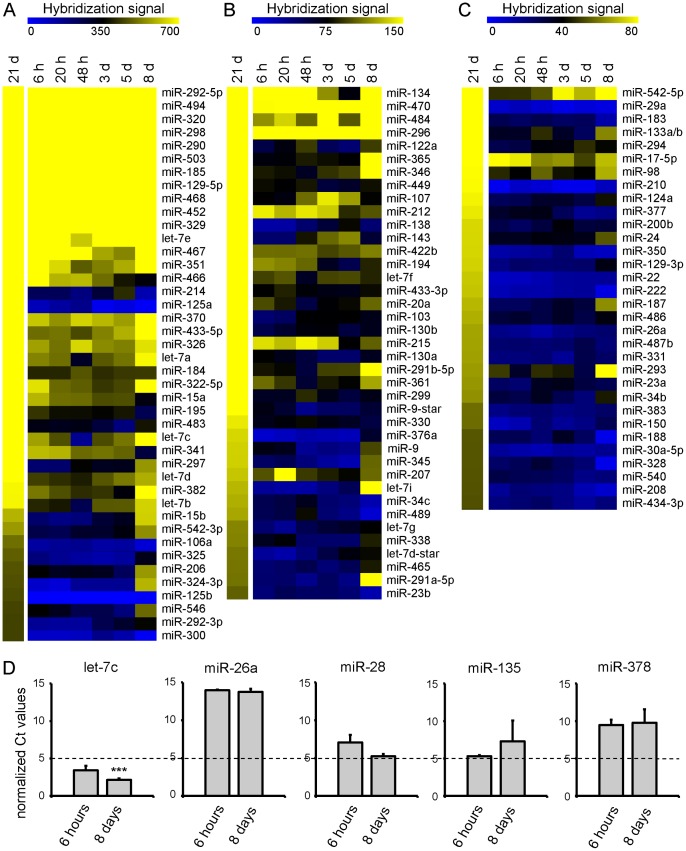
miRNA expression profiles in developing and mature hippocampal neurons. A–C) Heat maps showing average expression levels of miRNAs in hippocampal neurons during differentiation (6 h, 20 h, 48 h, 3 day and 5 days) compared to mature hippocampal neurons (21 days) in a blue (low relative expression) to yellow (high relative expression) scale. The selection criterion was an average expression value of >50. Using the following threshold signal values >400, 100–400 and 50–100, we subdivided the neuronal miRNAs in mature hippocampal neurons in respectively high (A), intermediate (B) and low expressing levels (C). The mean values were calculated from 4 independent experiments. One of the duplicate probes is shown in the figure D) Expression analysis of miRNAs by qPCR. Higher Ct value means low expression level of the miRNA. The expression of let-7c was changed with time, 6 hours versus 8 days. Single factor ANOVA was performed for statistical significance. Error bars represent standard deviation. ***p<0.0001.

Four independent batches of cultured hippocampal neurons were harvested at seven time points (6 hours, 20 hours, 48 hours, 3 days, 5 days, 8 days and 21 days) and total RNA/miRNA was isolated. These samples were then fluorescently labeled for the hybridization to locked nucleic-acid-based miRNA arrays containing 264 unique mouse miRNA probes and several control probes including non-protein coding RNAs, and some viral miRNAs in duplicate (as previously described by [60]). To ensure the reliability of the data, all of the hybridization experiments from seven time points were carried out eight times (four independent RNA samples and four sets of duplicate miRNA arrays were used) (Table S1). The fluorescent hybridization value for each spot was calculated by subtracting the background intensity from foreground intensity signals. The calculated values were highly consistent and reproducible within the same array and between experiments. Spots with verified background fluorescent signal values (negative controls, fluorescent signal <10) were filtered out of the data set. Using this threshold values a total of 210 miRNAs are expressed in mature hippocampal neurons at 21 days in culture (Table S1). Figure 2 shows 114 miRNAs with a fluorescent signal values 50 or higher. We subdivided this group according to high, intermediate and low expression levels using the threshold signal values >400, 100–400 and 50–100 (Fig. 2A–C, Table S2). To verify the miRNA array data set, we harvested hippocampal neurons at two time points (6 hours and 8 days), isolated total RNA, synthesized cDNA and performed quantitative PCR (qPCR) reactions on some selected miRNAs. Consistent with the miRNA array data, miRNAs with high (let7c) and low (miR-26a, miR-28, miR-135 and miR-378) expression levels can be detected by qPCR (Fig. 2D). Using the full miRNA array data set, we have identified several new miRNAs that showed significant expression and were not known to be present in the hippocampus. Some miRNAs, including miR-34, miR-125, miR-134, miR-138 were previously identified and characterized in the brain [61], [62], [63], [64], [65], [66], [67] (Fig. 2A–C). The founding member of the miRNA family let-7 and mir-125 are highly expressed in neurons consistent with previous data [18], [40], [41], [68] (Fig. 2A–C). Despite their high level of expression, the functional role of these conserved families in neuronal development is poorly characterized. Surprisingly, the expression of miR-132– one of the best-studied miRNA in the brain [66], [69] – is low in mature hippocampal neuron (fluorescent signal <50, Table S1) and therefore not indicated in Figure 2. On the other hand, we identified miRNAs that were previously shown to be preferentially expressed in neurons, including miR-124 [14], [20], [70], [71]. We also found some miRNAs, e.g., miR-23, miR-26 and miR-29 that were previously reported to be abundantly present in astrocytes [20]. These specific miRNAs are largely absent during the first days of neuronal development but present in more mature cultures at 21 days (Fig. 2A–C), which is most likely due to the presence of slow proliferating astrocytes in the neuronal cultures at this stage (Figure 1C–E).

### miRNAs expression profiling of developing hippocampal neurons in culture

**Figure 3:**
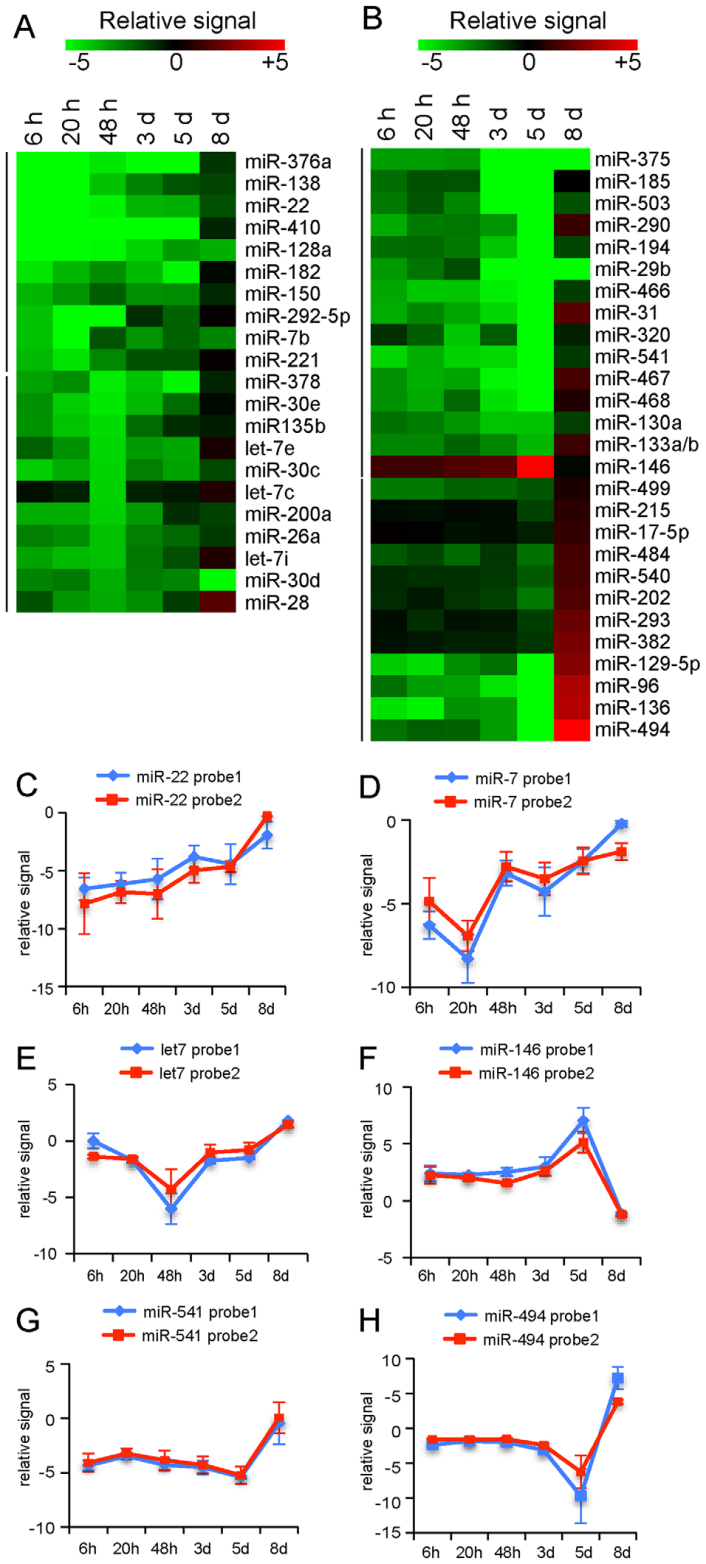
miRNA expression is regulated during early and late neuronal development. A–B) Heat maps showing relative expression levels of miRNAs in hippocampal neurons at 6 h, 20 h, 48 h, 3 day and 5 days compared to mature neurons (21 days) after plating in a green (negative fold-change) to red (positive fold-change) scale. Selection criteria; in all samples an absolute fold change value greater than or equal to 5 for negative fold changes P<0.01 and 1.5 for positive fold changes (P<0.05). Expression levels of miRNAs that are changed during early development (6 hours –48 hours) are shown in (A). Expression levels of miRNAs that are altered during late development (3 days –8 days) are shown in (B). (C–H) Graphs of relative signals (normalized fold changes) of miRNA expression during early neuronal development (C–E) and late neuronal differentiation (F–H). The blue and red lines indicate the two probes used to detect the expression of indicated miRNAs, which were spotted in duplicate on the LNA array.

We next compared the fold changes of miRNA expression in 21 day mature neurons with the various developmental stages at different time points (6 hours, 20 hours, 48 hours, 3 days, 5 days, 8 days) (Fig. 1), and found that 48 miRNAs were differentially expressed during development (Fig. 3, Table S2). Of these, 21 miRNAs were significantly altered during early development (Fig. 3A) and 27 miRNAs during late development (Fig. 3B). Almost all miRNAs (44/48) show strong upregulated expression at later stages of developing. Some miRNAs have low expression within the first 48 hours (stage 2 and 3) but the levels steady increase during later stages of neuronal development (Fig. 3A). For example, expression of miR-138 and miR-22 gradually increases over the different developmental time points (Fig. 3A, C). Other miRNAs show a clear drop in expression at specific developmental time points but are upregulated again at day 8 (Fig. 3B). For example, miR-7 shows a marked drop in expression at 20 hours in culture (Fig. 3D) and several let-7 miRNAs reveal decreased expression around 48 hours (Fig. 3A, E). These miRNAs could be involved in regulating specific processes during neuronal differentiation at stage 2 and 3, such as axon outgrowth, dendrite formation or dendritic spine development. Most of the miRNAs were differentially expressed with more than a 3-fold change between early (day 3 and 5) and late (day 8) developmental stage 4 (Fig. 3B, G). For example, the expression of miR-541 is stable for 5 days but at day 8 the levels are increased 5 fold (Fig. 3B, G). Five miRNAs, miR-129-5p, miR-96, miR-136 and miR-494, even show a ∼10 fold difference in expression between in vitro day 5 and day 8 (Fig. 3B, H). Although little is known about the role of these miRNAs in the mammalian brain, they could be involved in controlling synapse formation at this later stage of neuronal development. Of all the differentially expressed miRNAs during development (Fig. 3A, B), only 4 miRNAs, miR-29b, miR-30d, miR-146 and miR-375, showed decreased expression at day 8 (Fig. 3B, F). Together our experimental data indicate that a specific set of miRNAs is expressed in hippocampal neurons and that the expression of a large fraction of these miRNAs is altered during different developmental stages. Most of the miRNAs levels increase during neuronal differentiation.

### MiRNAs as modulators of axonal outgrowth

**Figure 4:**
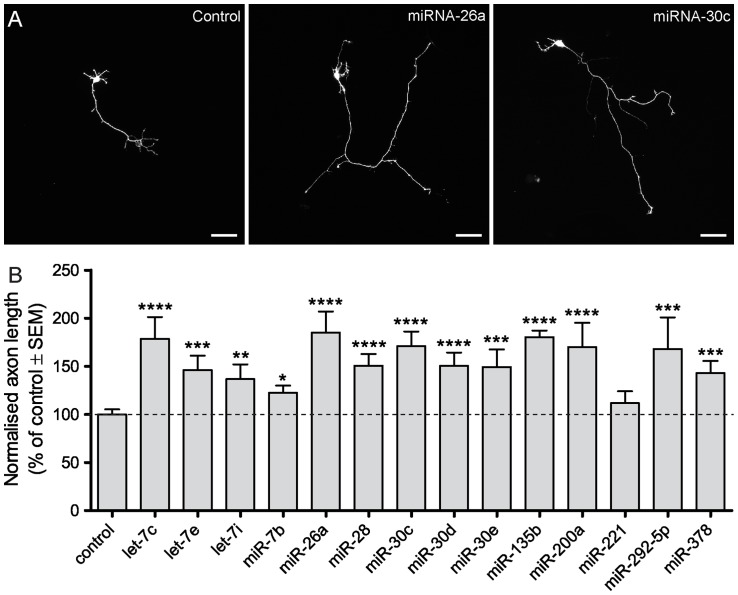
Sustained expression of subset of miRNAs modulates axonal outgrowth. A) Hippocampal dissociated neurons were co-transfected with GFP vector and miRIDIAN mimics for let-7c, let-7e, let-7i, miR-7b, miR-26a, miR-28, miR-30c, miR-30d, miR-30e, miR-135b, miR-200a, miR-221, miR-292-5p, miR-378 and miRNA. Three representative images of miRNA mimic transfected hippocampal neurons are shown. Scale bar, 50 μm. B) Graph represents measurement of total axon length. The average axon length of miRNA mimic expressing neurons is longer compared to control neurons (175.5 ± 10.2 μm). * p<0.01, ** p<0.001, *** p<0.0001, **** p<0.00001 in one way ANOVA with bonferroni correction for multiple testing.

The expression of most miRNAs is low at early stages during neuronal development (Fig. 2 and 3). However some specific miRNAs show reduced levels in stage 3 neurons, around the time of axonal outgrowth (20–48 h in culture). The 14 miRNAs that are strongly reduced at this stage of neuronal development are let-7b, let-7c, let-7e, let-7i, miR-26a, miR-28, miR-30c, miR-30d, miR-30e, miR-135b, miR-200a, miR-221, miR-292-5p and miR-378 (Fig. 3A, Table S1). Next, we investigated the effect of sustained expression of these specific miRNAs on neuronal development. Hippocampal neurons at 1 day were transfected with miRNA mimics and 4 days later analyzed for axon outgrowth. In almost all cases, the total axon length of neurons expressing miRNA mimics was markedly longer compared to control neurons (Fig. 4A). Quantification revealed that expression of 13 of the 14 miRNAs significantly accelerates axonal outgrowth during early neuronal development (Fig. 4B). These results suggest that sustained expression of this specific miRNA subset in stage 3 neurons can stimulate axonal outgrowth.

### Modulation of miRNAs expression by NMDA receptor-dependent synaptic plasticity

**Figure 5:**
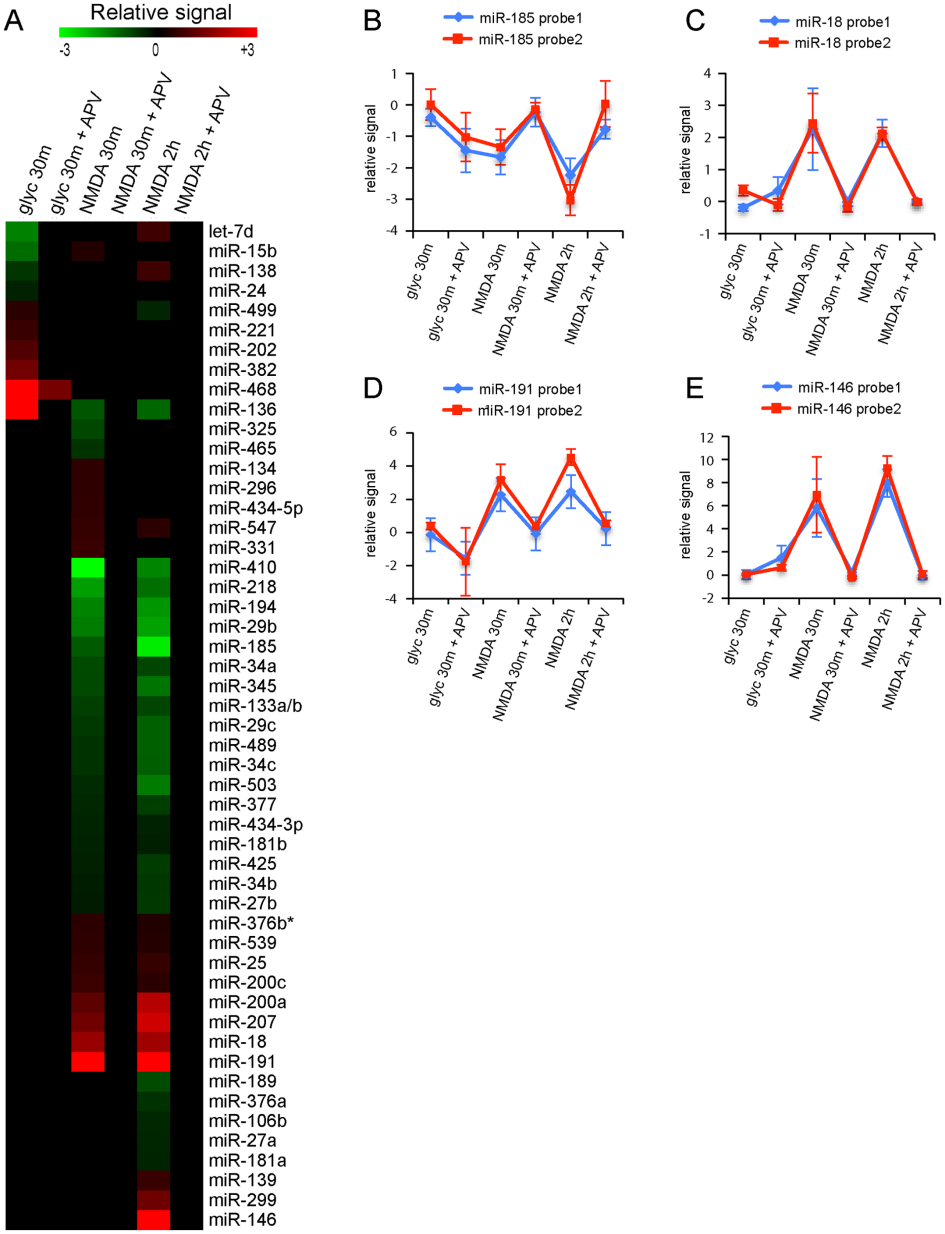
miRNA expression profiling following NMDA receptor-dependent synaptic plasticity. A) Heat map showing relative expression levels of miRNAs in mature hippocampal neurons (21 days) that are significantly changed following 5 min of 200 μM glycine treatment and recovered for 30 min (chemical LTP), 5 min of 50 μM NMDA treatment recovered for 30 min and 2 hours (chemical LTD) compared to non-treated neurons in a green (negative fold-change) to red (positive fold-change) scale. To control for the specificity of the treatments, all glycine en NMDA experiments were also performed in the presence of NMDA receptor inhibitor APV. Selection criteria; absolute fold change value greater than or equal to 1.4 with P<0.01 for NMDA P<0.05 for glycine and expression changes are blocked by APV. B–E) Graphs of relative signal (normalized fold changes) of miRNA expression following glycine and NMDA treatment compared to non-treated mature hippocampal neurons. The blue and red lines indicate the two probes used to detect the expression of indicated miRNAs, which were spotted in duplicate on the LNA array.

We next sought to identify miRNAs that associated with NMDAR-dependent synaptic plasticity in hippocampal neuron cultures induced by established chemical protocols. Bath application of cultures with 50 μM NMDA for 5 min induces “chemical” long-term depression (LTD) [72], [73], [74], [75], [76], while activation of synaptic NMDARs with 200 μM glycine for 5 min triggers “chemical” long-term potentiation (LTP) [77], [78], [79]. Microarray expression profiling was performed for chemical LTP-regulated miRNAs 30 minutes after glycine treatment and chemical LTD-regulated miRNAs 30 minutes and 2 hours after NMDA treatment using four independent batches of cultured hippocampal neurons. To control for the specificity of the treatments, all glycine and NMDA treatment experiments were also performed in the presence of 2R-amino-5-phosphonopentanoate (APV), an inhibitor of the NMDA receptor. The expression of 51 miRNAs was significantly altered after the induction of chemical LTP and/or LTD and this effect was blocked by APV (Fig. 5A–E, Table S2). The majority of these miRNAs (31/51) is present at low levels in mature hippocampal neurons (fluorescent signal <50, Table S1) and is not indicated in Figure 2. Of all modulated miRNAs, 10 miRNAs show expression changes after glycine treatment, while 34 miRNAs and 36 miRNAs show altered expression after 30 minutes and 2 hours after NMDA treatment, respectively (Fig. 5A). The expression of only one miRNA, miR-136 is strongly altered by both induction protocols, >4 fold increase after chemical LTP and >2 fold decrease after chemical LTD (Fig. 5A). Most miRNAs affected by glycine treatment (4 downregulated miRNAs and 6 upregulated miRNAs) have so far not been studied in neuronal cells. Many more miRNAs showed altered expression profiles after induction of chemical LTD –28 miRNAs were upregulated and 16 miRNAs were downregulated. For example, expression of miR-185 was decreased (Fig. 5B), whereas miR-18 and miR-191 showed an increase in expression after NMDA treatment (Fig. 5C, D). Most of the affected miRNAs have altered expression both at 30 minutes and 2 hours after application of NMDA (Fig. 5A), suggesting that induction of chemical LTD leads to long lasting changes in miRNA expression profiles (Fig. 5A). For example, the expression of miR-146 increased after 30 minutes and 2 hours of NMDA stimulation (∼8 fold increase, Fig. 5E). Only a few miRNAs were significantly altered only at 30 minutes (6 miRNAs) or at 2 hours (8 miRNAs) after NMDA treatment (Fig. 5A).

### Modulation of miRNAs expression by prolonged changes in global network activity

**Figure 6:**
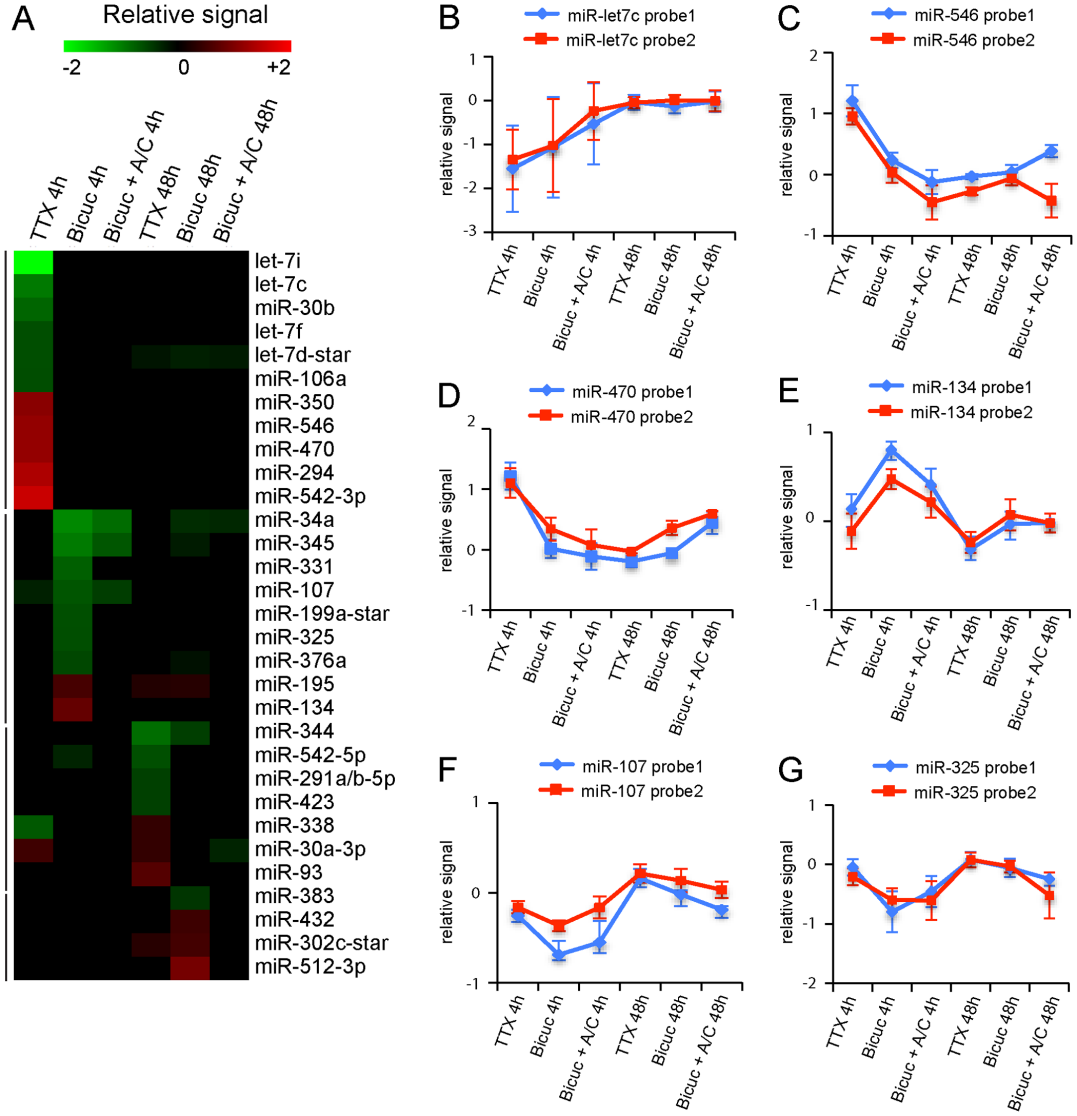
miRNA expression profiling following prolonged changes in global network activity. A) Heat map showing relative expression levels of miRNAs in mature hippocampal neurons (21 days) that are significantly changed following 4 hours and 48 hours 2 μM TTX and 4 hours and 48 hours 40 μM Bicuculine treatment compared to non-treated neurons in a green (negative fold-change) to red (positive fold-change) scale. To control for the specificity of the Bicuculine treatments, these experiments were also performed in the presence of APV and CNQX. Selection criteria were: absolute fold change value greater than or equal to 1.5 for 4 hour treatments and 1.4 for 48 hour treatments (P<0.05) and changes in Bicuculine expression are blocked by APV/CNQX. B–G) Graphs of relative signal (normalized fold changes) of miRNA expression following TTX and Bicuculine treatments compared to non-treated mature hippocampal neurons. The blue and red lines indicate the two probes used to detect the expression of indicated miRNAs, which were spotted in duplicate on the LNA array.

Homeostatic synaptic plasticity is a negative feedback mechanism that neurons use to offset excessive excitation or inhibition by adjusting their synaptic strengths. We next examined miRNA expression changes that are associated with prolonged changes in global network activity of hippocampal neuron cultures. To induce bidirectional homeostatic adaptations in mature cultured hippocampal neurons, we suppressed action potential-dependent synaptic activity with the voltage-gated sodium channels blocker tetrodotoxin (TTX, 2 μM, 48 h) or increased synaptic activity with the GABAA receptor antagonist bicuculline (Bicuc, 40 μM, 48 h) [54], [80]. Microarray expression profiling was performed for 4 hours and 48 hours after application of TTX or Bicuc using four independent batches of cultured hippocampal neurons. To control for the specificity of the treatments, the Bicuc treatment were also performed in the presence of APV and CNQX (6-cyano-7-nitroquinoxaline-2,3-dione). The expression of 31 miRNAs was significantly altered after TTX or Bicuc treatments (Fig. 6A–G, Table S2). Several of these miRNAs (13/31) are present at low levels in mature hippocampal neuron (fluorescent signal <50, Table S1). Of the 31 miRNAs, 18 miRNAs showed expression changes after TTX treatment and 13 miRNAs showed expression changes after Bicuc treatment (Fig. 6A). Interestingly, several of the miRNAs, including miR-34a, miR-345, miR-331, miR-325, miR-376a and miR-134 that were found altered after 4 hours Bicuc treatment were also identified in the NMDA treated samples (Fig. 5A). The change of this specific subset of miRNAs suggests that neuronal stimulation – at least in part – elicits a coordinated change in miRNA expression. Overall, two miRNA profiles appear within the homeostatic adaptation experiments – early miRNA expression changes and late miRNA expression effects. Most miRNAs have a significant altered expression only at one specific time point except for miR-30a-3p and miR-34a, the expression of which is increased after TTX treatment both at 4 hours and 48 hours (Fig. 6A). 7 miRNAs and 5 miRNAs show altered expression profiles after 48 hours TTX and Bicuc treatment, respectively. Most miRNAs showed altered expression profiles after 4 h TTX treatment –5 miRNAs were upregulated and 6 miRNAs were downregulated and 4 h Bicuc treatment –2 miRNAs were upregulated and 7 miRNAs are downregulated. For example, the expression of let-7c is decreased (Fig. 6B), whereas miR-546 and miR-470 showed an increase in expression after 4 hours TTX treatment (Fig. 6C, D). Moreover, expression of miR-134 was increased and miR-107 and miR-325 were decreased after 4 hours Bicuc treatment (Fig. 6E–G). These data indicate that specific miRNA expression patterns correlate with prolonged changes in neuronal activity.

## Discussion

This study describes an in-depth analysis of the hippocampal miRNA transcriptome during neuronal development (stage 2–4) and in mature neurons (stage 5) at the basal state and following robust neuronal activity changes. Using locked nucleic-acid-based miRNA arrays, we provide a systematic investigation of miRNA expression profiles of the classical Banker primary hippocampal neuron culture system. In total, we identified 210 miRNAs in mature hippocampal neurons. We found several new miRNAs that showed significant expression and were not known to be present in the hippocampus. We also identified members of the conserved miRNA family let-7 and miR-125 (the mammalian homologue of lin-4) and several other miRNAs that were previously shown to regulate specific developmental processes, including hippocampal axogenesis (miR-124) [71], dendrite formation (miR-124 and miR-134) [62], dendritic spine morphogenesis [64], [66], [81], spine structure (miR-134 and miR-138) [63], [64], and synaptic physiology (miR-34 and miR-125) [65], [66], [67]. Specific miRNAs reported to modulate cell proliferation and survival in other cells types are also abundantly expressed in hippocampal neurons, including anti-apoptotic miRNAs (miR-17-5p and miR-20a) [82] and senescence-related miRNAs (miR-290) [83]. Moreover, we show that expression levels of 51 of all tested miRNAs are regulated by NMDA receptor-dependent plasticity protocols and 31 miRNAs are induced by homeostatic plasticity, thereby demonstrating a feedback mechanism between neuronal signaling, miRNA levels and synaptic plasticity.

### Profiling miRNA expression during neuronal development

We have been able to identify developmental and stage-specific miRNAs, which might be associated with particular functions during neuronal development and differentiation. Using stringent criteria, 44 miRNA were differentially expressed with more than 5-fold changes during developmental stage 2–4. These miRNAs could be involved in regulating specific processes, such as axon outgrowth, dendrite formation, dendritic spine development or control of synapse formation at later stages of neuronal development. We observed that the majority of miRNAs expressed in the developing hippocampus are up-regulated during neuronal differentiation. Most miRNAs have low expression levels within the first 48 hours of development (stage 2 and 3) but increase during later stages of differentiation (Fig. 3A). For example, expression of miR-138 and miR-22 gradually increases over different time points. Only 4 miRNAs, miR-29b, miR-30d, miR-146 and miR-375, showed decreased expression at stage 4 (Fig. 3B, F). These down-regulated miRNAs have previously been reported in mammalian brain; miR-375 is involved in dendrite formation [33], miR-29 has been described as a regulator of synaptic morphology [84] and miR-30d is differentially expressed in the prefrontal cortex of individuals with schizophrenia and schizoaffective disorder [85]. Some other set of miRNAs showed altered expression levels at one particular developmental stage. In stage 3 neurons, around the time of axonal outgrowth (20–48 h in culture), 14 miRNA showed reduced expression (Fig. 3A). For example, miR-7 showed a marked drop in expression at 20 hours in culture and several let-7 miRNAs have decreased expression around 48 hours. Of all the miRNAs that show reduced expressions levels at early time points, we investigated the effect of sustained expression on neuronal development. We provide evidence that sustained expression of 13 of the 14 miRNAs during early neuronal development accelerates axonal outgrowth (Fig. 4). Together these data indicate that decreased expression of miRNAs during neuronal polarization is possibly involved in suppressing axon outgrowth at early stages of development. Therefore, the expression profiles established in this study provide new knowledge about translational regulation during hippocampal development and may be valuable in comparative studies of mRNA and protein expression profiles during early and late stages development [50], [51]. Additional studies are required to assess the precise role and downstream targets of the identified miRNA candidates.

Interestingly, miR-26a has been found to be a regulator of PTEN expression in gliomas [86]. Suppression of PTEN is well known to promote axon outgrowth [87], [88], suggesting that local modulation of PTEN protein levels by miR-26a could contribute to the observed axonal outgrowth phenotype. In cancer cells, upregulation of the miR-135 family was found associated with reduced expression of the adenomatous polyposis coli gene (APC) [89], [90]. APC is described to act on the cytoskeleton to direct axon polarization and outgrowth [91], [92], [93], and might provide the direct link between miR-135b and axon growth. Regardless of the precise miRNA targets involved, our data are consistent with several recent studies showing that overexpression of miRNAs can increase axon length in cortical neurons [94] and promote neurite extension from injured dorsal root ganglion (DRG) neurons [93], [95]. Interestingly, some miRNAs were found to be highly abundant in distal axons as compared with the cell bodies of primary sympathetic neurons [96], suggesting that some axonal miRNAs could regulate local protein synthesis during nerve growth [97].

### Profiling miRNA expression following synaptic plasticity

Neuronal activity controls protein synthesis in a variety of ways including the regulation of mRNA degradation or translational repression by miRNAs. A number of activity-regulated miRNAs have been identified and characterized in the hippocampus [6], [9], [98]. Regulation of miRNA expression by activity has been described for miR-125b [66], miR-132 [69], [81] and miR-134 [62]. Moreover, some miRNAs also associate with learning paradigms modeling drug addiction; miR-124, let-7d and miR-181 are found up-regulated in the nucleus accumbens in response to cocaine, and knockdown of each of these miRNAs influences conditioned place preference [99]. In agreement with these findings, we observe a number of miRNAs that are induced immediately following NMDA receptor-dependent plasticity and homeostatic plasticity. While some of these miRNAs have been described in other experiments, we identified several new activity-regulated miRNAs, such as miR-146, miR-185, miR-191 and miR-200a (NMDA receptor-dependent plasticity), and miR-107, miR-470 and miR-546 (homeostatic plasticity). Interestingly, most of the affected miRNAs have altered expression both at 30 minutes and 2 hours after the NMDA stimulation, demonstrating a stable change in miRNA levels for an extended period of time. The expression of a minority of miRNAs is altered at a specific time period. This is consistent with miRNA profiling studies using hippocampal slices [43], contextually conditioned mice [100], mice treated with electroconvulsive shock [42] or rats treated with high-frequency stimulation (HFS) [44]. Systematic analysis of miRNAs expression opens new possibilities to address the complexity of regulatory processes associated with neuronal development and changes in neuronal activity.

## Materials and Methods

### Ethics Statement

All animal experiments were performed in compliance with the guidelines for the welfare of experimental animals issued by the Federal Government of The Netherlands. All animal experiments were approved by the Animal Ethical Review Committee (DEC) of the Erasmus Medical Center and Utrecht University.

### Primary rat hippocampal neuron cultures, immunofluorescent staining and Western blotting

Primary hippocampal cultures were prepared from embryonic day 18 (E18) Wistar rat brains [57]. Cells were plated on coverslips coated with poly-L-lysine (30 μg/ml) and laminin (2 μg/ml) at a density of 100,000/well. Hippocampal cultures were grown in Neurobasal medium (NB) supplemented with B27, 0.5 mM glutamine, 12.5 μM glutamate and penicillin/streptomycin. For immunofluorescence stainings, neurons were fixed for 10 minutes with 4% paraformaldehyde/4% sucrose in phosphate-buffered saline (PBS) or 5 minutes with ice-cold 100% methanol/1 mM EGTA at −20°C, followed by 5 minutes with 4% paraformaldehyde/4% sucrose in PBS at room temperature. After fixation cells were washed two times in PBS for 30 min at room temperature, and incubated with primary antibodies in GDB buffer (0.2% BSA, 0.8 M NaCl, 0.5% Triton X-100, 30 mM phosphate buffer, pH 7.4) overnight at 4°C. Neurons were then washed three times in PBS for 5 min at room temperature and incubated with Alexa-conjugated secondary antibodies in GDB for 2 hr at room temperature and washed three times in PBS for 5 min. Slides were mounted using Vectashield mounting medium with DAPI (Vector laboratories). Images of fixed cells were collected with a Nikon Eclipse 80i equipped with Plan Apo VC 100× 1.4 N.A. and 60× 1.4 N.A. oil objectives and a CoolSNAP HQ2 camera (Roper Scientific).

For Western blotting cultured hippocampal neurons were harvested in 1× SDS sample buffer and boiled. Samples were loaded onto SDS–PAGE gels and subjected to western blotting on polyvinylidene difluoride membrane. Blots were blocked with 2% bovine serum albumin/0.05% Tween-20 in PBS and incubated with primary antibodies at 4°C overnight. Blots were washed with 0.05% Tween-20 in PBS, incubated with secondary antibodies and developed with enhanced chemiluminescent Western blotting substrate (Pierce). The following antibodies were used in this study: rabbit anti-MAP2 (Cell signaling) rabbit anti-ßIII-tubulin (Covance), mouse anti-tau (Millipore Bioscience Research Reagents), mouse anti-ßIII-tubulin (Covance), mouse anti-PSD-95 (NeuroMab), mouse anti-CaMKIIalpha (Sigma), mouse anti-GFAP (Sigma), Alexa Fluor 488- and Alexa Fluor 598-conjugated secondary antibodies (Invitrogen) and HRP-conjugated secondary antibodies (Dako).

### RNA extraction and Cy3-dye labeling

Total RNA was extracted using miRVana total RNA isolation kit (Ambion) according to manufacturer's instruction. RNA amount was measured using Nanodrop (Thermo Scientific). Total RNA was labeled with Cy3 containing dyes using the ULS aRNA labeling kit (Kreatech) according to the manufacturer's protocol in a total volume of 10 μl [60].

### Microarray preparation, hybridization, image analysis and data processing

LNA-modified oligonucleotide spotted glass slides (Exiqon) were processed using an automated hybridization station Tecan HS4800 pro hybridization station, which facilitates sample processing and hybridization standardization (as described by [60]). Microarrays with immobilized LNA-modified capture probes were hybridized at 60°C using microarray hybridization solution salt-based hybridization buffer (Ocimum Biosolutions). Hybridized slides were scanned in a Tecan LS Reloaded scanner. Data was extracted using Imagene 6.0 standard edition software. After background subtraction, the raw data were normalized using quantile normalization and processed for statistical analysis. During data extraction in Imagene, for each spot the quality was determined. Only qualified spots were used for statistical analysis. Constructing heatmaps and cluster analysis were done by using MultiExperiment Viewer (http://www.tm4.org/mev) [101].

After normalization the fluorescence intensities were averaged across all miRNA probes on each array. Each independent experiment comprised of at least two to four duplicate arrays. For replicate probes, the mean value was calculated to represent the miRNA fluorescent intensity value. To identify differentially expressed miRNAs, the data was analyzed using the unpaired t-test. P<0.05 was considered statistically significant. Fold changes described were calculated from the averages of the miRNA fluorescent intensity values of two to four independent experiments. Those miRNAs meeting a corrected cutoff with a p-value below 0.05 and with a fold change greater than 1.5 were considered differentially expressed. These and additional criteria as indicated in the main text were used to generate the lists of differentially expressed miRNA genes during neuronal differentiation, synaptic plasticity and activity.

### RNA isolation and quantitative real time PCR

Primary rat hippocampal neurons were plated at a density of 1000,000/well. Cells were harvested at two time points (6 hours and 8 days) with QIAzol lysis reagent and total RNA was extracted using the miRNeasy kit (Qiagen) according to the manufacturer's protocol. RNA quantity was determined using Nanodrop (Thermo Scientific) and equal amounts of each sample were used for first strand cDNA synthesis using universal cDNA synthesis kit (Exiqon). Quantitative PCR (qPCR) reactions were run on a 7900HT Real-Time PCR System (Applied Biosystems) using microRNA LNATM PCR primer sets and SYBR Green master mix (Exiqon). All samples were run in duplicates and the cycle threshold (Ct) values were determined using SDS software (Applied Biosystems). The expression levels of different miRNAs were estimated by normalizing to miR-124, the statistical significance was analyzed with single factor ANOVA and p<0.01 was considered as significant.

### Transfection of miRNA mimics in hippocampal neurons

C57BL/6 mouse pups of postnatal day 0–1 were decapitated and brains were rapidly removed in ice-cold dissection medium. Hippocampi were isolated, dissociated into single cells and cultured on acid-washed, poly-D-lysin-laminin coated coverslips at 37°C+5% CO2. On day in vitro 1 (DIV1), the neurons were co-transfected with a GFP vector and miRIDIAN mimics (Dharmacon) for let-7c, let-7e, let-7i, miR-7b, miR-26a, miR-28, miR-30c, miR-30d, miR-30e, miR-135b, miR-200a, miR-221, miR-292-5p, miR-378 and miR-control1 using Lipofectamine (Invitrogen). On DIV4 neurons were fixed with 4% paraformaldehyde and 4% sucrose in PBS. For immunohistochemistry the neurons were incubated with rabbit anti-GFP (Invitrogen) and mouse anti-ßIII-tubulin (Sigma) dissolved in buffer (0.2% gelatin, 0.6% triton X-100 in 33 mM NaHPO4 and 1.1 M NaCl). Images were taken using an Axioskop 2 EPI fluorescent microscope (Zeiss). The longest neurites were traced semimanually using NeuronJ plugin of ImageJ software. One-way ANOVAs with bonferroni correction were performed to statistically analyze the data.

## Supporting Information

Table S1 **Overview of the miRNA expression data at seven time points (6** **hours, 20** **hours, 48** **hours, 3** **days, 5** **days, 8** **days and 21** **days).** Four independent RNA samples and four sets of duplicate miRNA arrays were used for each time point, except for day 8. The fluorescent hybridization value for each spot was calculated by subtracting the background intensity from foreground intensity signals for each time point and batch (for example X6h1; neurons 6 hour in culture, batch 1). Spots with average fluorescent signal values >50 were use in Figure 2.
(XLSX)

Table S2 **Overview of the miRNA data presented in** **Figures** **3** **–** **6** **.** In this file the average hybridization signals and average fold changes are indicated.
(XLSX)

## References

[1] Malenka RC, Bear MF (2004) LTP and LTD: an embarrassment of riches. Neuron 44: 5–21. 15450156 10.1016/j.neuron.2004.09.012

[2] Massey PV, Bashir ZI (2007) Long-term depression: multiple forms and implications for brain function. Trends Neurosci 30: 176–184. 17335914 10.1016/j.tins.2007.02.005

[3] Neves G, Cooke SF, Bliss TV (2008) Synaptic plasticity, memory and the hippocampus: a neural network approach to causality. Nat Rev Neurosci 9: 65–75. 18094707 10.1038/nrn2303

[4] Li Q, Lee JA, Black DL (2007) Neuronal regulation of alternative pre-mRNA splicing. Nat Rev Neurosci 8: 819–831. 17895907 10.1038/nrn2237

[5] Steward O, Schuman EM (2003) Compartmentalized synthesis and degradation of proteins in neurons. Neuron 40: 347–359. 14556713 10.1016/s0896-6273(03)00635-4

[6] Swanger SA, Bassell GJ (2011) Making and breaking synapses through local mRNA regulation. Curr Opin Genet Dev 21: 414–421. 21530231 10.1016/j.gde.2011.04.002 PMC3149745

[7] Vo NK, Cambronne XA, Goodman RH (2010) MicroRNA pathways in neural development and plasticity. Curr Opin Neurobiol 20: 457–465. 20447821 10.1016/j.conb.2010.04.002

[8] Fineberg SK, Kosik KS, Davidson BL (2009) MicroRNAs potentiate neural development. Neuron 64: 303–309. 19914179 10.1016/j.neuron.2009.10.020

[9] Schratt G (2009) microRNAs at the synapse. Nat Rev Neurosci 10: 842–849. 19888283 10.1038/nrn2763

[10] Lim LP, Lau NC, Garrett-Engele P, Grimson A, Schelter JM, et al (2005) Microarray analysis shows that some microRNAs downregulate large numbers of target mRNAs. Nature 433: 769–773. 15685193 10.1038/nature03315

[11] Griffiths-Jones S, Saini HK, van Dongen S, Enright AJ (2008) miRBase: tools for microRNA genomics. Nucleic Acids Res 36: D154–158. 17991681 10.1093/nar/gkm952 PMC2238936

[12] Krichevsky AM, King KS, Donahue CP, Khrapko K, Kosik KS (2003) A microRNA array reveals extensive regulation of microRNAs during brain development. RNA 9: 1274–1281. 13130141 10.1261/rna.5980303 PMC1370491

[13] Miska EA, Alvarez-Saavedra E, Townsend M, Yoshii A, Sestan N, et al (2004) Microarray analysis of microRNA expression in the developing mammalian brain. Genome Biol 5: R68. 15345052 10.1186/gb-2004-5-9-r68 PMC522875

[14] Sempere LF, Freemantle S, Pitha-Rowe I, Moss E, Dmitrovsky E, et al (2004) Expression profiling of mammalian microRNAs uncovers a subset of brain-expressed microRNAs with possible roles in murine and human neuronal differentiation. Genome Biol 5: R13. 15003116 10.1186/gb-2004-5-3-r13 PMC395763

[15] Bak M, Silahtaroglu A, Moller M, Christensen M, Rath MF, et al (2008) MicroRNA expression in the adult mouse central nervous system. RNA 14: 432–444. 18230762 10.1261/rna.783108 PMC2248253

[16] Kosik KS (2006) The neuronal microRNA system. Nat Rev Neurosci 7: 911–920. 17115073 10.1038/nrn2037

[17] Liu NK, Wang XF, Lu QB, Xu XM (2009) Altered microRNA expression following traumatic spinal cord injury. Exp Neurol 219: 424–429. 19576215 10.1016/j.expneurol.2009.06.015 PMC2810508

[18] Ling KH, Brautigan PJ, Hahn CN, Daish T, Rayner JR, et al (2011) Deep sequencing analysis of the developing mouse brain reveals a novel microRNA. BMC Genomics 12: 176. 21466694 10.1186/1471-2164-12-176 PMC3088569

[19] Kim J, Krichevsky A, Grad Y, Hayes GD, Kosik KS, et al (2004) Identification of many microRNAs that copurify with polyribosomes in mammalian neurons. Proc Natl Acad Sci U S A 101: 360–365. 14691248 10.1073/pnas.2333854100 PMC314190

[20] Smirnova L, Grafe A, Seiler A, Schumacher S, Nitsch R, et al (2005) Regulation of miRNA expression during neural cell specification. Eur J Neurosci 21: 1469–1477. 15845075 10.1111/j.1460-9568.2005.03978.x

[21] Giraldez AJ, Cinalli RM, Glasner ME, Enright AJ, Thomson JM, et al (2005) MicroRNAs regulate brain morphogenesis in zebrafish. Science 308: 833–838. 15774722 10.1126/science.1109020

[22] Damiani D, Alexander JJ, O'Rourke JR, McManus M, Jadhav AP, et al (2008) Dicer inactivation leads to progressive functional and structural degeneration of the mouse retina. J Neurosci 28: 4878–4887. 18463241 10.1523/JNEUROSCI.0828-08.2008 PMC3325486

[23] Cuellar TL, Davis TH, Nelson PT, Loeb GB, Harfe BD, et al (2008) Dicer loss in striatal neurons produces behavioral and neuroanatomical phenotypes in the absence of neurodegeneration. Proc Natl Acad Sci U S A 105: 5614–5619. 18385371 10.1073/pnas.0801689105 PMC2291142

[24] Kawase-Koga Y, Otaegi G, Sun T (2009) Different timings of Dicer deletion affect neurogenesis and gliogenesis in the developing mouse central nervous system. Dev Dyn 238: 2800–2812. 19806666 10.1002/dvdy.22109 PMC2831750

[25] Huang T, Liu Y, Huang M, Zhao X, Cheng L (2010) Wnt1-cre-mediated conditional loss of Dicer results in malformation of the midbrain and cerebellum and failure of neural crest and dopaminergic differentiation in mice. J Mol Cell Biol 2: 152–163. 20457670 10.1093/jmcb/mjq008

[26] Davis TH, Cuellar TL, Koch SM, Barker AJ, Harfe BD, et al (2008) Conditional loss of Dicer disrupts cellular and tissue morphogenesis in the cortex and hippocampus. J Neurosci 28: 4322–4330. 18434510 10.1523/JNEUROSCI.4815-07.2008 PMC3844796

[27] Hebert SS, De Strooper B (2009) Alterations of the microRNA network cause neurodegenerative disease. Trends Neurosci 32: 199–206. 19268374 10.1016/j.tins.2008.12.003

[28] Konopka W, Kiryk A, Novak M, Herwerth M, Parkitna JR, et al (2012) MicroRNA loss enhances learning and memory in mice. J Neurosci 30: 14835–14842. 10.1523/JNEUROSCI.3030-10.2010 PMC6633640 21048142

[29] Li Q, Bian S, Hong J, Kawase-Koga Y, Zhu E, et al (2011) Timing specific requirement of microRNA function is essential for embryonic and postnatal hippocampal development. PLoS One 6: e26000. 21991391 10.1371/journal.pone.0026000 PMC3186801

[30] Bushati N, Cohen SM (2008) MicroRNAs in neurodegeneration. Curr Opin Neurobiol 18: 292–296. 18662781 10.1016/j.conb.2008.07.001

[31] Eacker SM, Dawson TM, Dawson VL (2009) Understanding microRNAs in neurodegeneration. Nat Rev Neurosci 10: 837–841. 19904280 10.1038/nrn2726 PMC4120241

[32] Gangaraju VK, Lin H (2009) MicroRNAs: key regulators of stem cells. Nat Rev Mol Cell Biol 10: 116–125. 19165214 10.1038/nrm2621 PMC4118578

[33] Abdelmohsen K, Hutchison ER, Lee EK, Kuwano Y, Kim MM, et al (2010) miR-375 inhibits differentiation of neurites by lowering HuD levels. Mol Cell Biol 30: 4197–4210. 20584986 10.1128/MCB.00316-10 PMC2937556

[34] Stefani G, Slack FJ (2008) Small non-coding RNAs in animal development. Nat Rev Mol Cell Biol 9: 219–230. 18270516 10.1038/nrm2347

[35] Provost P (2010) MicroRNAs as a molecular basis for mental retardation, Alzheimer's and prion diseases. Brain Res 1338: 58–66. 20347722 10.1016/j.brainres.2010.03.069 PMC2896967

[36] Forero DA, van der Ven K, Callaerts P, Del-Favero J (2010) miRNA genes and the brain: implications for psychiatric disorders. Hum Mutat 31: 1195–1204. 20725930 10.1002/humu.21344

[37] Liu NK, Xu XM (2011) MicroRNA in central nervous system trauma and degenerative disorders. Physiol Genomics 43: 571–580. 21385946 10.1152/physiolgenomics.00168.2010 PMC3110891

[38] Kan AA, van Erp S, Derijck AA, de Wit M, Hessel EV, et al (2012) Genome-wide microRNA profiling of human temporal lobe epilepsy identifies modulators of the immune response. Cell Mol Life Sci 69: 3127–3145. 22535415 10.1007/s00018-012-0992-7 PMC3428527

[39] McKiernan RC, Jimenez-Mateos EM, Bray I, Engel T, Brennan GP, et al (2012) Reduced mature microRNA levels in association with dicer loss in human temporal lobe epilepsy with hippocampal sclerosis. PLoS One 7: e35921. 22615744 10.1371/journal.pone.0035921 PMC3352899

[40] Wulczyn FG, Smirnova L, Rybak A, Brandt C, Kwidzinski E, et al (2007) Post-transcriptional regulation of the let-7 microRNA during neural cell specification. FASEB J 21: 415–426. 17167072 10.1096/fj.06-6130com

[41] Shinohara Y, Yahagi K, Kawano M, Nishiyori H, Kawazu C, et al (2011) miRNA profiling of bilateral rat hippocampal CA3 by deep sequencing. Biochem Biophys Res Commun 409: 293–298. 21575607 10.1016/j.bbrc.2011.05.004

[42] Eacker SM, Keuss MJ, Berezikov E, Dawson VL, Dawson TM (2011) Neuronal activity regulates hippocampal miRNA expression. PLoS One 6: e25068. 21984899 10.1371/journal.pone.0025068 PMC3184962

[43] Park CS, Tang SJ (2009) Regulation of microRNA expression by induction of bidirectional synaptic plasticity. J Mol Neurosci 38: 50–56. 18998061 10.1007/s12031-008-9158-3

[44] Wibrand K, Panja D, Tiron A, Ofte ML, Skaftnesmo KO, et al (2010) Differential regulation of mature and precursor microRNA expression by NMDA and metabotropic glutamate receptor activation during LTP in the adult dentate gyrus in vivo. Eur J Neurosci 31: 636–645. 20384810 10.1111/j.1460-9568.2010.07112.x PMC3791877

[45] Redell JB, Liu Y, Dash PK (2009) Traumatic brain injury alters expression of hippocampal microRNAs: potential regulators of multiple pathophysiological processes. J Neurosci Res 87: 1435–1448. 19021292 10.1002/jnr.21945 PMC5980641

[46] Yuan Y, Wang JY, Xu LY, Cai R, Chen Z, et al (2010) MicroRNA expression changes in the hippocampi of rats subjected to global ischemia. J Clin Neurosci 17: 774–778. 20080409 10.1016/j.jocn.2009.10.009

[47] Dotti CG, Sullivan CA, Banker GA (1988) The establishment of polarity by hippocampal neurons in culture. J Neurosci 8: 1454–1468. 3282038 10.1523/JNEUROSCI.08-04-01454.1988 PMC6569279

[48] Kaech S, Banker G (2006) Culturing hippocampal neurons. Nat Protoc 1: 2406–2415. 17406484 10.1038/nprot.2006.356

[49] Fletcher TL, Banker GA (1989) The establishment of polarity by hippocampal neurons: the relationship between the stage of a cell's development in situ and its subsequent development in culture. Dev Biol 136: 446–454. 2583372 10.1016/0012-1606(89)90269-8

[50] Mody M, Cao Y, Cui Z, Tay KY, Shyong A, et al (2001) Genome-wide gene expression profiles of the developing mouse hippocampus. Proc Natl Acad Sci U S A 98: 8862–8867. 11438693 10.1073/pnas.141244998 PMC37526

[51] Dabrowski M, Aerts S, Van Hummelen P, Craessaerts K, De Moor B, et al (2003) Gene profiling of hippocampal neuronal culture. J Neurochem 85: 1279–1288. 12753086 10.1046/j.1471-4159.2003.01753.x

[52] Luscher C, Xia H, Beattie EC, Carroll RC, von Zastrow M, et al (1999) Role of AMPA receptor cycling in synaptic transmission and plasticity. Neuron 24: 649–658. 10595516 10.1016/s0896-6273(00)81119-8

[53] Sala C, Piech V, Wilson NR, Passafaro M, Liu G, et al (2001) Regulation of dendritic spine morphology and synaptic function by Shank and Homer. Neuron 31: 115–130. 11498055 10.1016/s0896-6273(01)00339-7

[54] Ehlers MD (2003) Activity level controls postsynaptic composition and signaling via the ubiquitin-proteasome system. Nat Neurosci 6: 231–242. 12577062 10.1038/nn1013

[55] Jaworski J, Kapitein LC, Gouveia SM, Dortland BR, Wulf PS, et al (2009) Dynamic microtubules regulate dendritic spine morphology and synaptic plasticity. Neuron 61: 85–100. 19146815 10.1016/j.neuron.2008.11.013

[56] Kapitein LC, Schlager MA, Kuijpers M, Wulf PS, van Spronsen M, et al (2010) Mixed microtubules steer dynein-driven cargo transport into dendrites. Curr Biol 20: 290–299. 20137950 10.1016/j.cub.2009.12.052

[57] Kapitein LC, Yau KW, Hoogenraad CC (2010) Microtubule dynamics in dendritic spines. Methods Cell Biol 97: 111–132. 20719268 10.1016/S0091-679X(10)97007-6

[58] Haber M, Zhou L, Murai KK (2006) Cooperative astrocyte and dendritic spine dynamics at hippocampal excitatory synapses. J Neurosci 26: 8881–8891. 16943543 10.1523/JNEUROSCI.1302-06.2006 PMC6675342

[59] Jones EV, Cook D, Murai KK (2012) A neuron-astrocyte co-culture system to investigate astrocyte-secreted factors in mouse neuronal development. Methods Mol Biol 814: 341–352. 22144317 10.1007/978-1-61779-452-0_22

[60] Pothof J, Verkaik NS, van IW, Wiemer EA, Ta VT, et al (2009) MicroRNA-mediated gene silencing modulates the UV-induced DNA-damage response. EMBO J 28: 2090–2099. 19536137 10.1038/emboj.2009.156 PMC2718280

[61] Yu JY, Chung KH, Deo M, Thompson RC, Turner DL (2008) MicroRNA miR-124 regulates neurite outgrowth during neuronal differentiation. Exp Cell Res 314: 2618–2633. 18619591 10.1016/j.yexcr.2008.06.002 PMC2702206

[62] Fiore R, Khudayberdiev S, Christensen M, Siegel G, Flavell SW, et al (2009) Mef2-mediated transcription of the miR379-410 cluster regulates activity-dependent dendritogenesis by fine-tuning Pumilio2 protein levels. EMBO J 28: 697–710. 19197241 10.1038/emboj.2009.10 PMC2647767

[63] Schratt GM, Tuebing F, Nigh EA, Kane CG, Sabatini ME, et al (2006) A brain-specific microRNA regulates dendritic spine development. Nature 439: 283–289. 16421561 10.1038/nature04367

[64] Siegel G, Obernosterer G, Fiore R, Oehmen M, Bicker S, et al (2009) A functional screen implicates microRNA-138-dependent regulation of the depalmitoylation enzyme APT1 in dendritic spine morphogenesis. Nat Cell Biol 11: 705–716. 19465924 10.1038/ncb1876 PMC3595613

[65] Agostini M, Tucci P, Steinert JR, Shalom-Feuerstein R, Rouleau M, et al (2011) microRNA-34a regulates neurite outgrowth, spinal morphology, and function. Proc Natl Acad Sci U S A 108: 21099–21104. 22160706 10.1073/pnas.1112063108 PMC3248521

[66] Edbauer D, Neilson JR, Foster KA, Wang CF, Seeburg DP, et al (2010) Regulation of synaptic structure and function by FMRP-associated microRNAs miR-125b and miR-132. Neuron 65: 373–384. 20159450 10.1016/j.neuron.2010.01.005 PMC5018398

[67] Muddashetty RS, Nalavadi VC, Gross C, Yao X, Xing L, et al (2011) Reversible inhibition of PSD-95 mRNA translation by miR-125a, FMRP phosphorylation, and mGluR signaling. Mol Cell 42: 673–688. 21658607 10.1016/j.molcel.2011.05.006 PMC3115785

[68] Juhila J, Sipila T, Icay K, Nicorici D, Ellonen P, et al (2011) MicroRNA expression profiling reveals miRNA families regulating specific biological pathways in mouse frontal cortex and hippocampus. PLoS One 6: e21495. 21731767 10.1371/journal.pone.0021495 PMC3120887

[69] Mellios N, Sugihara H, Castro J, Banerjee A, Le C, et al (2011) miR-132, an experience-dependent microRNA, is essential for visual cortex plasticity. Nat Neurosci 14: 1240–1242. 21892155 10.1038/nn.2909 PMC3183341

[70] Krichevsky AM, Sonntag KC, Isacson O, Kosik KS (2006) Specific microRNAs modulate embryonic stem cell-derived neurogenesis. Stem Cells 24: 857–864. 16357340 10.1634/stemcells.2005-0441 PMC2605651

[71] Sanuki R, Onishi A, Koike C, Muramatsu R, Watanabe S, et al (2011) miR-124a is required for hippocampal axogenesis and retinal cone survival through Lhx2 suppression. Nat Neurosci 14: 1125–1134. 21857657 10.1038/nn.2897

[72] Beattie EC, Carroll RC, Yu X, Morishita W, Yasuda H, et al (2000) Regulation of AMPA receptor endocytosis by a signaling mechanism shared with LTD. Nat Neurosci 3: 1291–1300. 11100150 10.1038/81823

[73] Colledge M, Snyder EM, Crozier RA, Soderling JA, Jin Y, et al (2003) Ubiquitination regulates PSD-95 degradation and AMPA receptor surface expression. Neuron 40: 595–607. 14642282 10.1016/s0896-6273(03)00687-1 PMC3963808

[74] Ehlers MD (2000) Reinsertion or degradation of AMPA receptors determined by activity-dependent endocytic sorting. Neuron 28: 511–525. 11144360 10.1016/s0896-6273(00)00129-x

[75] Kameyama K, Lee HK, Bear MF, Huganir RL (1998) Involvement of a postsynaptic protein kinase A substrate in the expression of homosynaptic long-term depression. Neuron 21: 1163–1175. 9856471 10.1016/s0896-6273(00)80633-9

[76] Lee HK, Kameyama K, Huganir RL, Bear MF (1998) NMDA induces long-term synaptic depression and dephosphorylation of the GluR1 subunit of AMPA receptors in hippocampus. Neuron 21: 1151–1162. 9856470 10.1016/s0896-6273(00)80632-7

[77] Fortin DA, Davare MA, Srivastava T, Brady JD, Nygaard S, et al (2010) Long-term potentiation-dependent spine enlargement requires synaptic Ca2+-permeable AMPA receptors recruited by CaM-kinase I. J Neurosci. 30: 11565–11575. 10.1523/JNEUROSCI.1746-10.2010 PMC2943838 20810878

[78] Lu W, Man H, Ju W, Trimble WS, MacDonald JF, et al (2001) Activation of synaptic NMDA receptors induces membrane insertion of new AMPA receptors and LTP in cultured hippocampal neurons. Neuron 29: 243–254. 11182095 10.1016/s0896-6273(01)00194-5

[79] Park M, Salgado JM, Ostroff L, Helton TD, Robinson CG, et al (2006) Plasticity-induced growth of dendritic spines by exocytic trafficking from recycling endosomes. Neuron 52: 817–830. 17145503 10.1016/j.neuron.2006.09.040 PMC1899130

[80] Hoogenraad CC, Feliu-Mojer MI, Spangler SA, Milstein AD, Dunah AW, et al (2007) Liprinalpha1 degradation by calcium/calmodulin-dependent protein kinase II regulates LAR receptor tyrosine phosphatase distribution and dendrite development. Dev Cell 12: 587–602. 17419996 10.1016/j.devcel.2007.02.006

[81] Wayman GA, Davare M, Ando H, Fortin D, Varlamova O, et al (2008) An activity-regulated microRNA controls dendritic plasticity by down-regulating p250GAP. Proc Natl Acad Sci U S A 105: 9093–9098. 18577589 10.1073/pnas.0803072105 PMC2449370

[82] Aranha MM, Santos DM, Xavier JM, Low WC, Steer CJ, et al (2010) Apoptosis-associated microRNAs are modulated in mouse, rat and human neural differentiation. BMC Genomics 11: 514. 20868483 10.1186/1471-2164-11-514 PMC2997008

[83] Pitto L, Rizzo M, Simili M, Colligiani D, Evangelista M, et al (2009) miR-290 acts as a physiological effector of senescence in mouse embryo fibroblasts. Physiol Genomics 39: 210–218. 19723773 10.1152/physiolgenomics.00085.2009

[84] Lippi G, Steinert JR, Marczylo EL, D'Oro S, Fiore R, et al (2011) Targeting of the Arpc3 actin nucleation factor by miR-29a/b regulates dendritic spine morphology. J Cell Biol 194: 889–904. 21930776 10.1083/jcb.201103006 PMC3207289

[85] Perkins DO, Jeffries CD, Jarskog LF, Thomson JM, Woods K, et al (2007) microRNA expression in the prefrontal cortex of individuals with schizophrenia and schizoaffective disorder. Genome Biol 8: R27. 17326821 10.1186/gb-2007-8-2-r27 PMC1852419

[86] Huse JT, Brennan C, Hambardzumyan D, Wee B, Pena J, et al (2009) The PTEN-regulating microRNA miR-26a is amplified in high-grade glioma and facilitates gliomagenesis in vivo. Genes Dev 23: 1327–1337. 19487573 10.1101/gad.1777409 PMC2701585

[87] Christie KJ, Webber CA, Martinez JA, Singh B, Zochodne DW (2010) PTEN inhibition to facilitate intrinsic regenerative outgrowth of adult peripheral axons. J Neurosci 30: 9306–9315. 20610765 10.1523/JNEUROSCI.6271-09.2010 PMC6632469

[88] Sun F, Park KK, Belin S, Wang D, Lu T, et al (2011) Sustained axon regeneration induced by co-deletion of PTEN and SOCS3. Nature 480: 372–375. 22056987 10.1038/nature10594 PMC3240702

[89] Holleman A, Chung I, Olsen RR, Kwak B, Mizokami A, et al (2011) miR-135a contributes to paclitaxel resistance in tumor cells both in vitro and in vivo. Oncogene 30: 4386–4398. 21552288 10.1038/onc.2011.148 PMC3572709

[90] Nagel R, le Sage C, Diosdado B, van der Waal M, Oude Vrielink JA, et al (2008) Regulation of the adenomatous polyposis coli gene by the miR-135 family in colorectal cancer. Cancer Res 68: 5795–5802. 18632633 10.1158/0008-5472.CAN-08-0951

[91] Purro SA, Ciani L, Hoyos-Flight M, Stamatakou E, Siomou E, et al (2008) Wnt regulates axon behavior through changes in microtubule growth directionality: a new role for adenomatous polyposis coli. J Neurosci 28: 8644–8654. 18716223 10.1523/JNEUROSCI.2320-08.2008 PMC2832753

[92] Shi SH, Cheng T, Jan LY, Jan YN (2004) APC and GSK-3beta are involved in mPar3 targeting to the nascent axon and establishment of neuronal polarity. Curr Biol 14: 2025–2032. 15556865 10.1016/j.cub.2004.11.009

[93] Zhou S, Shen D, Wang Y, Gong L, Tang X, et al (2012) microRNA-222 targeting PTEN promotes neurite outgrowth from adult dorsal root ganglion neurons following sciatic nerve transection. PLoS One 7: e44768. 23028614 10.1371/journal.pone.0044768 PMC3441418

[94] Zhang Y, Ueno Y, Liu XS, Buller B, Wang X, et al (2013) The MicroRNA-17–92 Cluster Enhances Axonal Outgrowth in Embryonic Cortical Neurons. J Neurosci 33: 6885–6894. 23595747 10.1523/JNEUROSCI.5180-12.2013 PMC3657758

[95] Strickland IT, Richards L, Holmes FE, Wynick D, Uney JB, et al (2011) Axotomy-induced miR-21 promotes axon growth in adult dorsal root ganglion neurons. PLoS One 6: e23423. 21853131 10.1371/journal.pone.0023423 PMC3154476

[96] Natera-Naranjo O, Aschrafi A, Gioio AE, Kaplan BB (2010) Identification and quantitative analyses of microRNAs located in the distal axons of sympathetic neurons. RNA 16: 1516–1529. 20584895 10.1261/rna.1833310 PMC2905752

[97] Han L, Wen Z, Lynn RC, Baudet ML, Holt CE, et al (2011) Regulation of chemotropic guidance of nerve growth cones by microRNA. Mol Brain 4: 40. 22051374 10.1186/1756-6606-4-40 PMC3217933

[98] Ashraf SI, Kunes S (2006) A trace of silence: memory and microRNA at the synapse. Curr Opin Neurobiol 16: 535–539. 16962314 10.1016/j.conb.2006.08.007

[99] Chandrasekar V, Dreyer JL (2009) microRNAs miR-124, let-7d and miR-181a regulate cocaine-induced plasticity. Mol Cell Neurosci 42: 350–362. 19703567 10.1016/j.mcn.2009.08.009

[100] Kye MJ, Neveu P, Lee YS, Zhou M, Steen JA, et al (2011) NMDA mediated contextual conditioning changes miRNA expression. PLoS One 6: e24682. 21931811 10.1371/journal.pone.0024682 PMC3171446

[101] Saeed AI, Sharov V, White J, Li J, Liang W, et al (2003) TM4: a free, open-source system for microarray data management and analysis. Biotechniques 34: 374–378. 12613259 10.2144/03342mt01

